# eQTpLot: a user-friendly R package for the visualization of colocalization between eQTL and GWAS signals

**DOI:** 10.1186/s13040-021-00267-6

**Published:** 2021-07-17

**Authors:** Theodore G. Drivas, Anastasia Lucas, Marylyn D. Ritchie

**Affiliations:** 1 Division of Human Genetics, Children’s Hospital of Philadelphia, Philadelphia, PA USA; 2 Department of Genetics, Perelman School of Medicine, University of Pennsylvania, Philadelphia, PA USA; 3 Institute for Biomedical Informatics, Perelman School of Medicine, University of Pennsylvania, Philadelphia, PA USA

**Keywords:** GWAS, eQTL, Colocalization, Visualization

## Abstract

**Background:**

Genomic studies increasingly integrate expression quantitative trait loci (eQTL) information into their analysis pipelines, but few tools exist for the visualization of colocalization between eQTL and GWAS results. Those tools that do exist are limited in their analysis options, and do not integrate eQTL and GWAS information into a single figure panel, making the visualization of colocalization difficult.

**Results:**

To address this issue, we developed the intuitive and user-friendly R package eQTpLot. eQTpLot takes as input standard GWAS and cis-eQTL summary statistics, and optional pairwise LD information, to generate a series of plots visualizing colocalization, correlation, and enrichment between eQTL and GWAS signals for a given gene-trait pair. With eQTpLot, investigators can easily generate a series of customizable plots clearly illustrating, for a given gene-trait pair: 1) colocalization between GWAS and eQTL signals, 2) correlation between GWAS and eQTL *p*-values, 3) enrichment of eQTLs among trait-significant variants, 4) the LD landscape of the locus in question, and 5) the relationship between the direction of effect of eQTL signals and the direction of effect of colocalizing GWAS peaks. These clear and comprehensive plots provide a unique view of eQTL-GWAS colocalization, allowing for a more complete understanding of the interaction between gene expression and trait associations.

**Conclusions:**

eQTpLot provides a unique, user-friendly, and intuitive means of visualizing eQTL and GWAS signal colocalization, incorporating novel features not found in other eQTL visualization software. We believe eQTpLot will prove a useful tool for investigators seeking a convenient and customizable visualization of eQTL and GWAS data colocalization.

**Availability and implementation:**

the eQTpLot R package and tutorial are available at https://github.com/RitchieLab/eQTpLot

## Background

Non-protein-coding genetic variants make up the majority of statistically significant associations identified by genome wide association studies (GWAS). As these variants typically do not have obvious consequences for gene function, it can be difficult to map their effects to specific genes. To address this issue, genomic studies have increasingly begun to integrate expression quantitative trait loci (eQTL) information into their analysis pipelines, with the thought that non-coding variants might be exerting their effects on patient phenotypes through the modulation of expression levels of nearby genes. Through this approach, indirect evidence for causality can be obtained if a genetic locus significantly associated with candidate gene expression levels is found to colocalize with a genetic locus significantly associated with the phenotype of interest.

A number of excellent tools have been developed to discover and analyze colocalization between eQTL and GWAS association signals [1–8], but few packages provide the necessary tools to visualize these colocalizations in an intuitive and informative way. LocusCompare [8] allows for the side-by-side visualization of eQTL and GWAS signal colocalization, but does not visually integrate this data. LocusZoom [9] produces a single plot integrating linkage disequilibrium (LD) information and GWAS data, but does not consider eQTL data. Furthermore, no colocalization visualization tool exists that takes into account the direction of effect of an eQTL with relation to the direction of effect of colocalizing GWAS signals.

For these reasons, we developed eQTpLot, an R package for the intuitive visualization of colocalization between eQTL and GWAS signals. In its most basic implementation, eQTpLot takes standard GWAS summary data, formatted as one might obtain from a GWAS analysis in PLINK [10], and cis-eQTL data, formatted as one might download directly from the GTEx portal [11], to generate a series of customizable plots clearly illustrating, for a given gene-trait pair: 1) colocalization between GWAS and eQTL signals, 2) correlation between GWAS and eQTL *p*-values, 3) enrichment of eQTLs among trait-significant variants, 4) the LD landscape of the locus in question, and 5) the relationship between the directions of effect of eQTL signals and colocalizing GWAS peaks. These clear and comprehensive plots provide a unique view of eQTL-GWAS colocalization, allowing for a more complete understanding of the interaction between gene expression and trait associations. We believe eQTpLot will prove a useful tool for investigators seeking a convenient and robust visualization of genomic data colocalization.

## Implementation

eQTpLot was developed in R version 4.0.0 and depends on a number of packages for various aspects of its implementation (biomaRt, dplyr, GenomicRanges, ggnewscale, ggplot2, ggplotfy, ggpubr, gridExtra, Gviz, LDheatmap, patchwork) [12–21]. The software is freely available on GitHub (https://github.com/RitchieLab/eQTpLot) and can be downloaded for use at the command line, or in any R-based integrated development environment, such as RStudio. Example data and a complete tutorial on the use of eQTpLot and its various features have also been made available on GitHub.

At a minimum, eQTpLot requires two input files, imported into R as data frames: one of GWAS summary statistics (as might be obtained from a standard associations study as completed in PLINK [10]) and one of cis-eQTL summary statistics (as might be downloaded directly from the GTEx portal at gtexportal.org [11]). Table 1 summarizes the formatting parameters of the two required input files and of the two optional input files. Additionally, there are many options that can be specified to generate variations of the main eQTpLot, as discussed below. Table 2 shows the complete list of command line arguments that can be passed to eQTpLot, with descriptions of their use.

**Table 1:** Description of required and optional input data frames for eQTpLot

Required Input Data Frames		
**GWAS.df**, a data frame, one row per SNP, with columns as one might obtain from a genome-wide association study performed in PLINK using either the --logistic or --linear flags		
***Column Name***	***Data type***	***Description***
**CHR**	Integer	Chromosome for SNP (sex chromosomes coded numerically)
**BP**	Integer	Chromosomal position for each SNP, in base pairs
**SNP**	Character	Variant ID (such as dbSNP ID “rs...”. (*Note: naming scheme must be the same as what is used in the* ***eQTL.df*** *to ensure proper SNP matching*)
**P**	Numeric	P-value for the SNP from GWAS analysis
**BETA**	Numeric	Beta for SNP from GWAS analysis
**PHE** *(Optional)*	Character	Name of the phenotype for which the GWAS data refers. This column is optional and is useful if your **GWAS.df** contains data for multiple phenotypes, such as one might obtain from a PheWAS. If **GWAS.df** does not contain a “PHE” column, eQTpLot will assume all the supplied GWAS data is for a single phenotype, with a name to be specified with the “trait” argument.
**eQTL.df**, a data frame, one row per SNP, with columns as one might download directly from the GTEx Portal in .csv format		
***Column Name***	***Data type***	***Description***
**SNP.Id**	Character	Variant ID (such as dbSNP ID “rs...”. (*Note: naming scheme must be the same as what is used in the* ***GWAS.df*** *to ensure proper matching*).
**Gene.Symbol**	Character	Gene symbol to which the eQTL expression data refers (*Note: gene symbol must match entries in* ***Genes.df*** *to ensure proper matching*)
**P.value**	Numeric	P-value for the SNP from eQTL analysis
**NES**	Numeric	Normalized effect size for the SNP from eQTL analysis (Per GTEx, defined as the slope of the linear regression, and is computed as the effect of the alternative allele relative to the reference allele in the human genome reference.
**Tissue**	Character	Tissue type to which the eQTL pvalue/NES refer (*Note:* ***eQTL.df*** *can contain multiple tissue types*)
**N** *(Optional)*	Numeric	The number of samples used to calculate the p-value and NES for the eQTL data. This value is used if performing a MultiTissue or PanTissue analysis with the option CollapseMethod set to “meta” for a simple sample size weighted meta-analysis.
**Optional Input Data Frames**		
**Genes.df**, an optional data frame, one row per gene, with the following columns (*Note: eQTpLot automatically loads a default* ***Genes.df*** *containing information for most protein-coding genes for genomic builds hg19 and hg38, but you may wish to specify our own* ***Genes.df*** *data frame if your gene of interest is not included in the default data frame, or if your eQTL data uses a different gene naming scheme (for example, Gencode ID instead of gene symbol))*		
*Column Name*	*Data type*	*Description*
**Gene**	Character	Gene symbol/name (*Note: gene naming scheme must match entries in* ***eQTL.df*** *to ensure proper matching*)
**CHR**	Integer	Chromosome the gene is on (*Note: do not include a “chr” prefix, and sex chromosomes should be coded numerically*)
**Start**	Integer	Base pair coordinate of the beginning of the gene (*Note: this should be the smaller of the two values between* ***Start*** *and* ***Stop****)*
**Stop**	Integer	Base pair coordinate of the end of the gene (*Note: this should be the larger of the two values between* ***Start*** *and* ***Stop****)*
**Build**	*Character,* “hg19” *or* “hg38”	The genome build (either hg19 or hg38) for the location data
**LD.df**, an optional data frame of SNP linkage data, one row per SNP pair, with columns as one might obtain from a PLINK linkage disequilibrium analysis using the PLINK --r2 option. (*Note: If no* ***LD.df*** *is supplied, eQTpLot will plot data without LD information)*		
*Column Name*	*Data type*	*Description*
**BP_A**	Integer	Base pair position of the first variant in the LD pair
**SNP_A**	Character	Variant ID of the first variant in the LD pair (*Note: only variants that also appear in the* ***GWAS.df*** *SNP column will be used for LD analysis*)
**BP_B**	Integer	Base pair position of the second variant in the LD pair
**SNP_B**	Character	Variant ID of the second variant in the LD pair (*Note: only SNPs that also appear in the* ***GWAS.df*** *SNP column will be used for LD analysis*)
**R2**	Numeric	Squared correlation measure of linkage between the two variants

**Table 2:** Description of required and optional arguments for eQTpLot

**Required Arguments**	
***Argument***	***Description***
**eQTL.df**	A data frame of eQTL summary statistic data, as defined in Table 1
**GWAS.df**	A data frame of GWAS summary statistic data, as defined in Table 1
**gbuild**	***Default value is “hg19”***. The genome build, in quotes, to use for fetching genomic information for the genome track (panel B). This build should match the genome build used for “CHR” and “BP” in the **GWAS.df**. Currently the only compatible options are “hg19” and “hg38”
**gene**	The name/symbol of the gene to analyze, in quotes (*Note: gene name must match an entry in* ***Genes.df*** *for the specified* ***gbuild***)
**sigpvalue_eQTL**	***Default value is 0.05***. The significance threshold to use for eQTL data (variants with an eQTL *p*-value larger than this threshold will be excluded from the analysis)
**sigpvalue_GWAS**	***Default value is 5e-8***. The significance threshold to use for GWAS data (this value will be used for a horizontal line in plot A, and to define GWAS significant/non-significant variants for the eQTL enrichment plot).
**tissue**	***Default value is “all”.*** The tissue name, in quotes, to use for analysis. **eQTL.df** entries will be filtered to contain only data on this tissue. If this parameter is set to “all”, eQTpLot will pick the smallest eQTL p-value for each SNP across all tissues for a PanTissue analysis. Alternatively, a list of tissue names can be supplied (in the format c(“tissue1”, “tissue2”, …) to perform a PanTissue analysis on just these tissues. (*Note: the tissue name must match at least one entry in the* ***eQTL.df*** *Tissue column*)
**trait**	The name of the GWAS phenotype to analyze, in quotes. If all the data in **GWAS.df** is for a single phenotype and no PHE column is present, this argument will be used as the name for the analyzed phenotype. If **GWAS.df** contains information on multiple phenotypes, as specified in the optional **GWAS.df** PHE column, this parameter will be used to filter in **GWAS.df** entries for only this phenotype.
**Optional arguments**	
***Argument***	***Description***
**Genes.df**	A data frame of gene coordinates, as defined in Table 1
**LD.df**	A data frame of pairwise linkage data, as defined in Table 1
**congruence**	***Default value is FALSE***. If set to TRUE, variants with congruent and incongruent effects will be plotted separately, as described below.
**genometrackheight**	***Default value is 2*** Used to set the height of the genome track panel (B). Gene-dense regions may require more plotting space, whereas gene-sparse regions may look better with less plotting space.
**getplot**	***Default value is TRUE***. If set to FALSE, eQTpLot will not display the generated plot in the viewport.
**LDcolor**	***Only used if LD.df is supplied. Default value is “color”***. For the LDheatmap panel, the heatmap will be filled using a grayscale palate if this argument is set to “black”, or with a full color palate if this argument is set to “color”.
**LDmin**	***Only used if LD.df is supplied. Default value is 10***. For the LDheatmap panel, only variants that are in LD (with R^2^ > **R2min**) with at least this many other variants will be displayed. This parameter can be useful to thin the number of variants being plotted in the LDheatmap.
**leadSNP**	***Only used if LD.df is supplied.*** This parameter is used to specify the lead SNP ID, in quotes, to use for plotting LD information in the P-P plots. The specified variant must be present in both the **GWAS.df** and **LD.df** data frames.
**NESeQTLRange**	the maximum and minimum limits in the format c (min,max), to display for the NES value in **eQTL.df**. The default setting will adjust the size scale automatically to fit the displayed data, whereas specifying the limits will keep them consistent between plots.
**R2min**	***Only used if LD.df is supplied. Default value is 0.1***. The threshold for R^2^ to use when selecting LD data from **LD.df**. Variant pairs with R^2^ < **R2min** will not be included in the analysis.
**range**	***Default value is 200***. The range, in kB, to extend the analysis window on either side of the gene of interest, as defined by the **Start** and **Stop** points for the specified **gene** in **Genes.df**.
**res**	***Default value is 300***. The resolution, in dpi, for the output plot image
**saveplot**	***Default value is TRUE.*** If set to TRUE, eQTpLot will save the generated plot in the working directory with the name “**gene**.**trait**.**tissue**. Congreunce_Info.LD_Info.eQTpLot.png”, using the variables and arguments provided.
**wi**	***Default value is 12 if LD.df is not supplied, 14 if LD.df is supplied.*** The width of the output plot image, in inches. The height of the plot is calculated from this argument as well to maintain the appropriate aspect ratio.
**xlimd**	used to manually adjust the x axis maximum for the P-P plot, if needed
**ylima**	used to manually adjust the y axis maximum in plot A, if needed
**ylimd**	used to manually adjust the y axis maximum for the P-P plot, if needed
**CollapsMethod**	***Default value is “min”.*** This parameter dictates the method used to collapse eQTL *p*-values and NES across tissues if a MultiTissue or PanTissue analysis is specified. If set to “min” the *p*-value and NES from the tissue with the smallest p-value for each variant will be selected. If set to “median” or “mean” the median or mean p-value and NES for each variant, across all specified tissues, will be selected. If set to “meta” eQTpLot will perform a simple sample-size-weighted meta-analysis [22, 23] of the *p*-values across all specified tissues. *(NOTE: If “meta” is specified,* ***eQTL.df*** *should include a column with header “N” indicating the number of samples used to derive the given eQTL data. If no column N is present, eQTpLot will give the user the option to complete a meta-analysis assuming equal sample sizes for all tissues, which may lead to inaccurate results. Also note that if “meta” is specified, no meta-analyzed NES will be computed, and all variants will be displayed as the same size in the main eQTpLot figure.)*
**Gene.List**	***Default value is FALSE.*** If set to TRUE, this parameter will output the Pearson correlation between eQTL and GWAS p-values for a given tissue across a user-supplied list of genes, ordered by significance. No plots will be generated. If the user sets the parameter tissue to “all,” or to a list of tissues, eQTpLot will collapse the eQTL data for these tissues by variant, using the method specified by the parameter **CollapseMethod**. This may be a useful parameter to obtain a very simple bird’s-eye view of the genes at a locus whose expression is most closely correlated to a relevant GWAS signal for a given trait.
**Tissue.List**	***Default value is FALSE.*** If set to TRUE, this parameter will output the Pearson correlation between eQTL and GWAS p-values for a given gene across a user-supplied list of tissues, ordered by significance. No plots will be generated. If the user sets the parameter **tissue** to “all,” eQTpLot will consider each tissue included in **eQTL.df**. This may be a useful parameter to obtain a very simple bird’s-eye view of the tissues in which a given gene’s expression is most closely tied to a relevant GWAS signal for a given trait.

## Results and discussion

In its simplest implementation, eQTplot takes as input two data frames, one of GWAS summary data and the other of eQTL summary data, with the user specifying the name of the gene to be analyzed, the GWAS trait to be analyzed (useful if the GWAS data contains information on multiple associations, as one might obtain from a Phenome-wide Association Study (PheWAS)), and the tissue type to use for the eQTL analysis. Using these inputs, eQTpLot generates a series of plots intuitively illustrating the colocalization of GWAS and eQTL signals in chromosomal space, and the enrichment of and correlation between the candidate gene eQTLs and trait-significant variants. Additional parameters and data can be supplied, such as pairwise variant LD information, allowing for an even more comprehensive visualization of the interaction between eQTL and GWAS data within a given genomic locus.

One major implementation feature that sets eQTpLot apart from other eQTL visualization software is the option to divide eQTL/GWAS variants into groups based on their directions of effect. If the argument **congruence** is set to TRUE, all variants are divided into two groups: congruous, or those with the same direction of effect on gene expression and the GWAS trait (e.g., a variant that is associated with increased expression of the candidate gene and an increase in the GWAS trait), and incongruous, or those with opposite directions of effect on gene expression and the GWAS trait (e.g., a variant that is associated with increased expression of the candidate gene but a decrease in the GWAS trait). The division between congruous and incongruous variants provides a more nuanced view of the relationship between gene expression level and GWAS associations – a variant associated with increased expression of a candidate gene and an increase in a given GWAS trait would seem to be operating through different mechanisms than a variant that is similarly associated with increased expression of the same candidate gene, but a decrease in the same GWAS trait. eQTpLot intuitively visualizes these differences as described below. This distinction also serves to illuminate important underlying biologic difference between different gene-trait pairs, discriminating between genes that appear to suppress a particular phenotype and those that appear to promote it.

Another important feature of eQTpLot that is not found in other eQTL visualization software is the ability to specify a PanTissue or MultiTissue eQTL visualization. In some instances, it may be of interest to visualize a variant’s effect on candidate gene expression across multiple tissue types, or even across all tissues. Such analyses can be accomplished by setting the argument **tissue** to a list of tissues contained within **eQTL.df** (e.g. c(“Adipose_Subcutaneous”, “Adipose_Visceral”)) for a MultiTissue analysis, or by setting the argument **tissue** to “all” for a PanTissue analysis. In a PanTissue analysis, eQTL data across all tissues contained in **eQTL.df** will be collapsed, by variant, into a single pan-tissue eQTL; a similar approach is used in a MultiTissue analysis, but in this case eQTL data will be collapsed, by variant, across only the specified tissues. The method by which eQTpLot collapses eQTL data can be specified with the argument **CollapseMethod**, which accepts as input one of four options – “min,” “median,” “mean,” or “meta.” By setting **CollapseMethod** to “min” (the default), for each variant the tissue with the smallest eQTL *p*-value will be selected, such that each variant’s most significant eQTL effect, agnostic of tissue, can be visualized. Setting the parameter to “median” or “mean” will visualize the median or mean *p*-value and NES value for each SNP across all specified tissues. Lastly, setting **CollapseMethod** to “meta” will perform a simple sample-size-weighted meta-analysis (i.e. a weighted Z-test) [22, 23] for each variant across all specified tissues, visualizing the resultant p-value for each variant. It should be noted that this meta-analysis method requires a sample size for each eQTL entry in **eQTL.df**, which should be supplied in an optional column “N.” If sample size numbers are not readily available (as may be the case if directly downloading cis-eQTL data from the GTEx portal), eQTpLot gives the user the option to presume that all eQTL data is derived from identical sample sizes across all tissues – this approach may of course yield inaccurate estimates of a variant’s effect in meta-analysis, but may be useful to the user.

What follows is a description of the process used to generate each of the plots produced by eQTpLot, along with a series of use examples to both demonstrate the utility of eQTpLot, and to highlight some of the many options that can be combined to generate different outputs. For these examples we have analyzed a subset of data from our recently-published analysis of quantitative laboratory traits in the UK Biobank [24] – these summary statistics are available in full at https://ritchielab.org/publications/supplementary-data/ajhg-cilium, and the subset of summary data used for our example analyses can be downloaded from the eQTpLot GitHub page such that the reader may experiment with eQTpLot with the pre-supplied data.

### Generation of the main eQTL-GWAS Colocalization plot

**Fig. 1:**
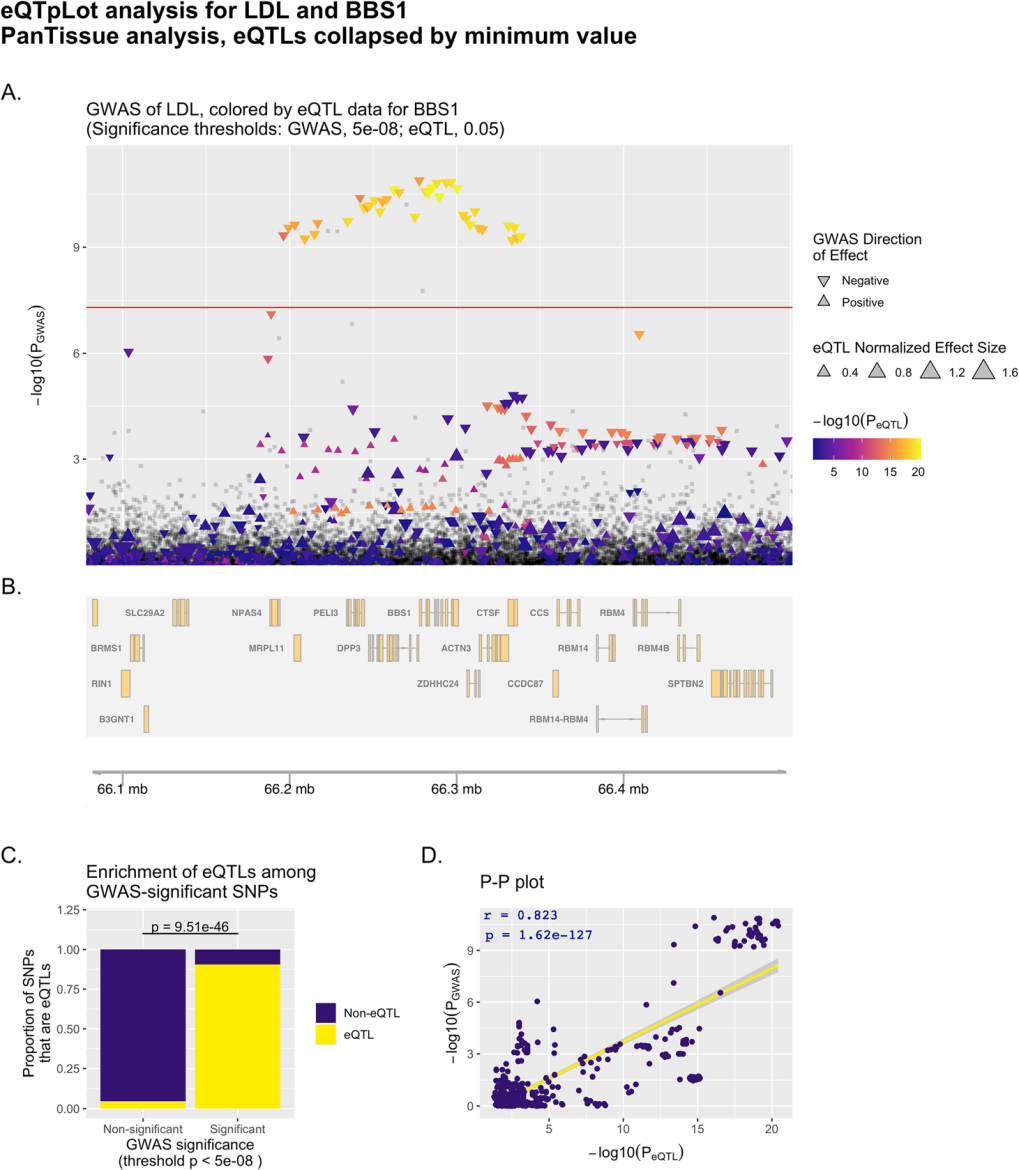
Example eQTpLot for LDL cholesterol and the gene *BBS1*. eQTpLot was used to generate a series of plots illustrating the colocalization between eQTLs for the gene *BBS1* and a GWAS signal for the LDL cholesterol trait on chromosome 11 using a PanTissue approach as described in example 1. Panel **A** shows the locus of interest, containing the *BBS1* gene, with chromosomal space indicated along the horizontal axis. The position of each point on the vertical axis corresponding to the *p*-value of association for that variant with the LDL trait, while the color scale for each point corresponds to the magnitude of that variant’s p-value for association with *BBS1* expression. The directionality of each triangle corresponds to the GWAS direction of effect, while the size of each triangle corresponds to the NES for the eQTL data. The default genome-wide p-value significance threshold for the GWAS analysis, 5e-8, is depicted with a horizontal red line. Panel **B** displays the genomic positions of all genes within the LOI. Panel **C** depicts the enrichment of *BBS1* eQTLs among GWAS-significant variants, while panel **D** depicts the correlation between P_GWAS_ and P_eQTL_ for *BBS1* and the LDL trait, with the computed Pearson correlation coefficient (r) and *p*-value (p) displayed on the plot

**Fig. 2:**
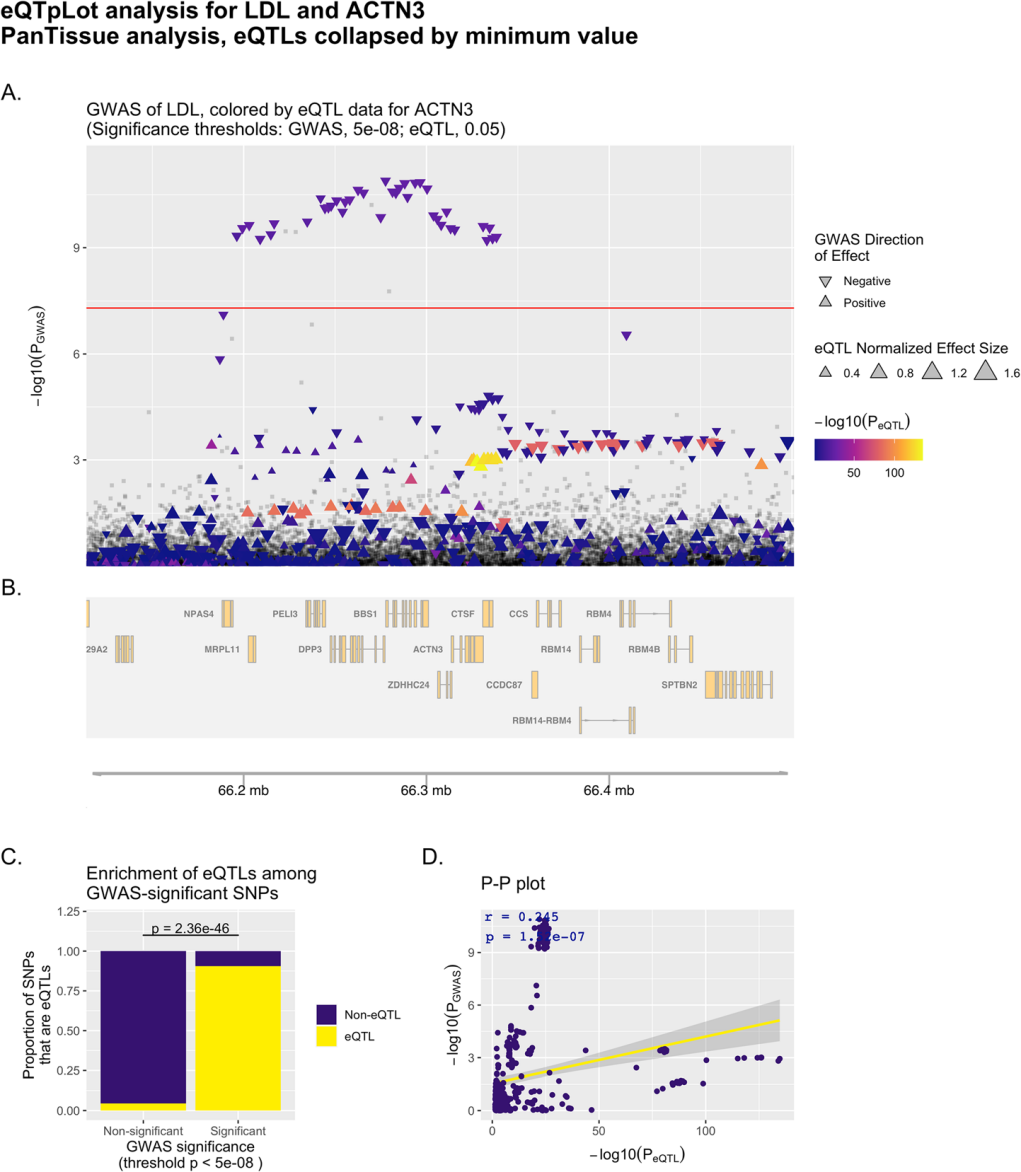
Example eQTpLot for LDL cholesterol and the gene *ACTN3*. eQTpLot was used to generate a series of plots illustrating the colocalization between eQTLs for the gene *ACTN3* and a GWAS signal for the LDL cholesterol trait on chromosome 11 using a PanTissue approach as described in example 1. Panel **A** shows the locus of interest, containing the *ACTN3* gene, with chromosomal space indicated along the horizontal axis. The position of each point on the vertical axis corresponding to the *p*-value of association for that variant with the LDL trait, while the color scale for each point corresponds to the magnitude of that variant’s p-value for association with *ACTN3* expression. The directionality of each triangle corresponds to the GWAS direction of effect, while the size of each triangle corresponds to the NES for the eQTL data. The default genome-wide *p*-value significance threshold for the GWAS analysis, 5e-8, is depicted with a horizontal red line. Panel **B** displays the genomic positions of all genes within the LOI. Panel **C** depicts the enrichment of *ACTN3* eQTLs among GWAS-significant variants, while panel **D** depicts the correlation between P_GWAS_ and P_eQTL_ for *ACTN3* and the LDL trait, with the computed Pearson correlation coefficient (r) and p-value (p) displayed on the plot

**Fig. 3:**
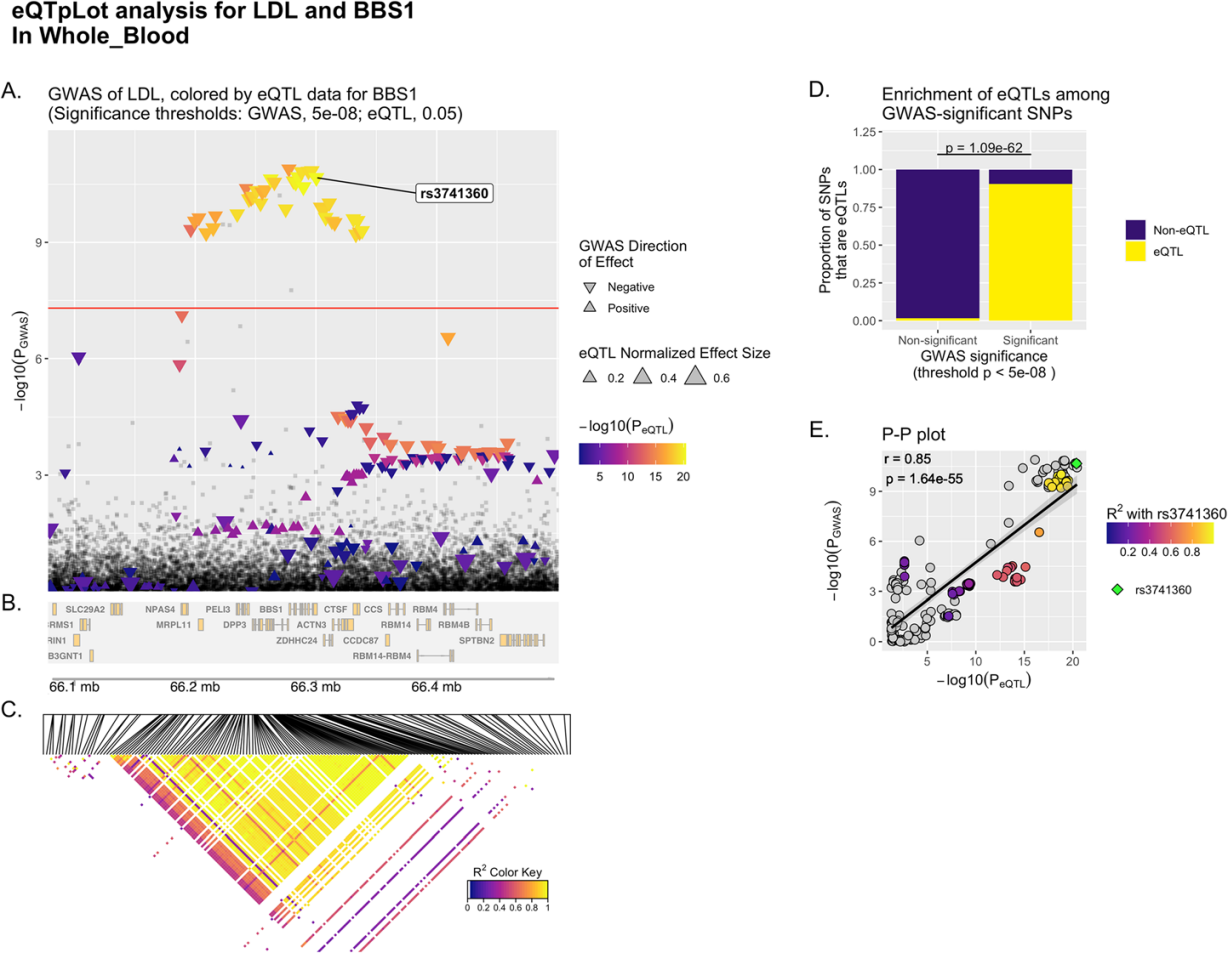
Example eQTpLot for LDL cholesterol and the gene *BBS1*, incorporating LD data. eQTpLot was used to generate a series of plots illustrating the colocalization between eQTLs for the gene *BBS1* and a GWAS signal for the LDL cholesterol trait on chromosome 11 as described in example 2, specifically within the tissue “Whole_Blood” and with the inclusion of LD data. Panels **A**, **B**, and **D** are generated identically to Figure panels 1A, 1B, and 1C respectively. Panel **C** depicts a heatmap of LD information of all *BBS1* eQTL variants, displayed in the same chromosomal space as panels **A** and **B** for ease of reference. Panel **E** depicts the correlation between P_GWAS_ and P_eQTL_ for *BBS1* and the LDL trait, similar to panel 1D, only here a lead variant, rs3741360, is identified (by default the upper-right-most variant on the P-P plot), with all other variants plotted using a color scale corresponding to their squared coefficient of linkage correlation with this lead variant. For reference, the same lead variant is also labelled in panel **A**

**Fig. 4:**
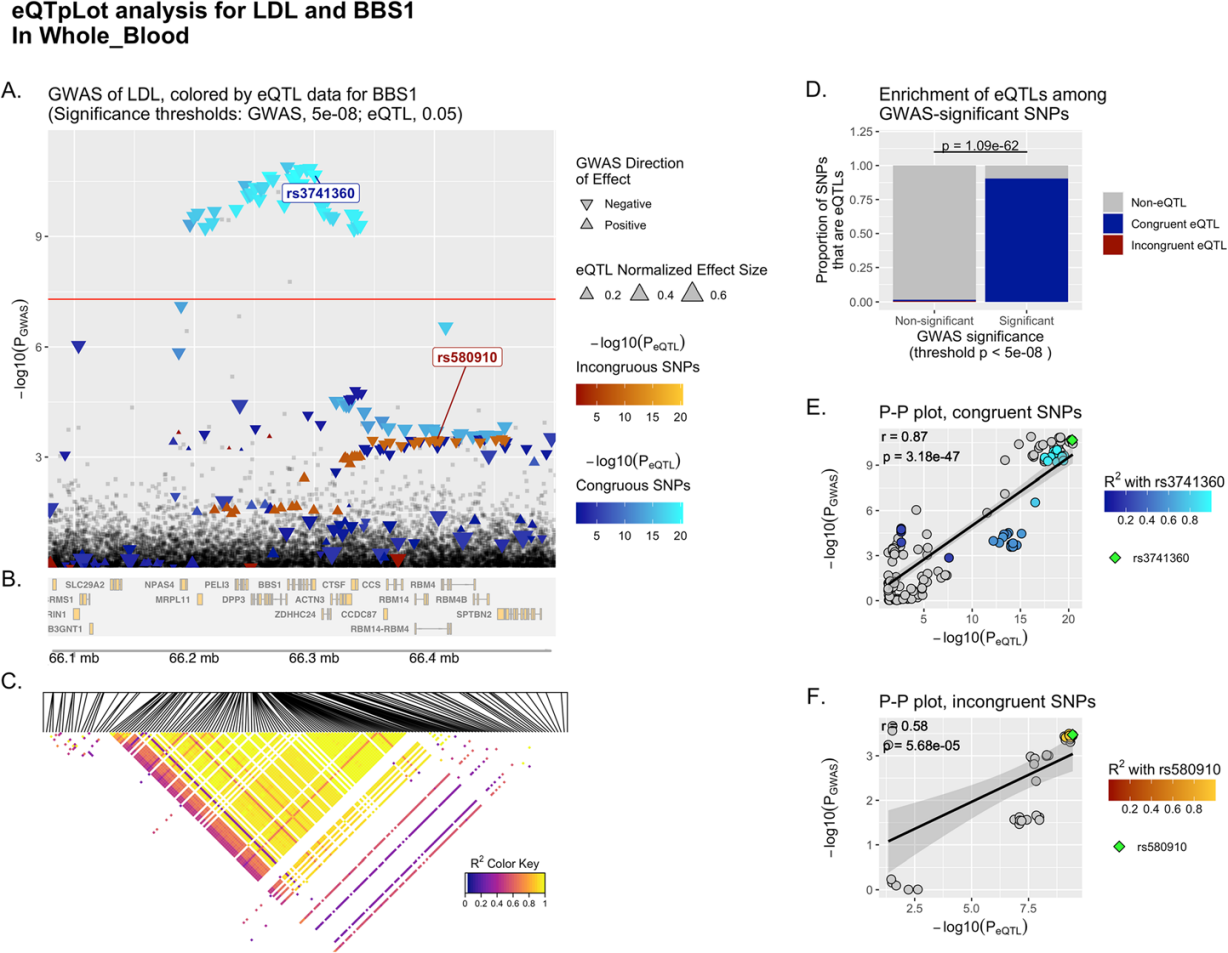
Example eQTpLot for LDL cholesterol and the gene *BBS1*, discriminating between congruous and incongruous variants. eQTpLot was used to generate a series of plots illustrating the colocalization between eQTLs for the gene *BBS1* and a GWAS signal for the LDL cholesterol trait on chromosome 11 as described in example 3, with an analysis identical to that described for Fig. 3, but with the additional discrimination between variants with congruous and incongruous directions of effect. Panel **A** is generated identically to panel 1A and 3A, only instead of using a single color scale, variants with congruous effects are plotted using a blue color scale, while variants with incongruous effects are plotted using a red color scale. Panels **B**-**D** are identical to panels 3B-D. Panel **E** and **F** both represent P-P plots, generated similarly to the P-P plot in panel 3E. For panel **E**, however, the analysis is confined only to variants with congruous directions of effect, while for panel **F** the analysis includes only variants with incongruous directions of effect. A lead variant is indicated in both panels **E** anf **F**, and both are also labeled in panel **A**

To generate the main eQTL-GWAS Colocalization Plot (Figs. 1A, 2A, 3A, 4A), a locus of interest (LOI) is defined to include the target gene’s chromosomal coordinates (as listed in **Genes.df,** for the indicated **gbuild**, for the user-specified **gene**), along with a range of flanking genome (specified with the argument **range**, with a default value of 200 kilobases on either side of the gene). GWAS summary statistics from **GWAS.df** are filtered to include only variants that fall within the LOI. The variants are then plotted in chromosomal space along the horizontal axis, with the inverse log of the *p*-value of association with the specified GWAS trait (P_GWAS_) plotted along the vertical axis, as one would plot a standard GWAS Manhattan plot. The GWAS significance threshold, **sigpvalue_GWAS** (default value 5e-8), is depicted with a red horizontal line.

Within this plot, variants that lack eQTL data for the target gene in **eQTL.df **(or for which the eQTL p-value (P_eQTL_) does not meet the specified significance threshold, **sigpvalue_eQTL** (default value 0.05)) are plotted as grey squares. On the other hand, variants that act as eQTLs for the target gene (with P_eQTL_ < **sigpvalue_eQTL**) are plotted as colored triangles, with a color gradient corresponding to the inverse magnitude of P_eQTL_. As noted above, an analysis can be specified to differentiate between variants with congruous versus incongruous effects on the GWAS trait and candidate gene expression levels – if this is the case, variants with congruous effects will be plotted using a blue color scale, while variants with incongruous effects will be plotted using a red color scale (as seen in Fig. 4A). The size of each triangle corresponds to the eQTL normalized effect size (NES) for each variant, while the directionality of each triangle is set to correspond to the direction of effect for the variant on the GWAS trait.

A depiction of the genomic positions of all genes within the LOI is generated below the plot using the package Gviz (Figs. 1B, 2B, 3B, 4B) [12]. If LD data is supplied, in the form of **LD.df**, a third panel illustrating the LD landscape of eQTL variants within the LOI is generated using the package LDheatmap (Fig. 3C, 4C) [20]. To generate this panel, **LD.df** is filtered to contain only eQTL variants that appear in the plotted LOI, and to include only variant pairs that are in LD with each other with R^2^ > **R2min** (default value of 0.1). This dataset is further filtered to include only variants that are in LD (with R^2^ > **R2min**) with at least a certain number of other variants (user-defined with the argument **LDmin**, default value of 10). These filtering steps are useful in paring down the number of variants to be plotted in the LDheatmap, keeping the most informative variants and reducing the time needed to generate the eQTpLot. A heatmap illustrating the pairwise linkage disequilibrium of the final filtered variant set is subsequently generated below the main eQTL-GWAS Colocalization Plot, with a fill scale corresponding to R^2^ for each variant pair. The location of each variant in chromosomal space is indicated at the top of the heatmap, using the same chromosomal coordinates as displayed in panels A and B.

### Generation of the eQTL enrichment plot

For variants within the LOI with P_GWAS_ less than the specified GWAS significance threshold, **sigpvalue_GWAS**, the proportion that are also eQTLs for the gene of interest (with P_eQTL_ < **sigpvalue_eQTL**) are calculated and plotted, and the same is done for variants with P_GWAS_ > **sigpvalue_GWAS**, (Fig. 1C, 2C, 3D, 4D). Enrichment of candidate gene eQTLs among GWAS-significant variants is determined by Fisher’s exact test. If an analysis differentiating between congruous and incongruous variants is specified, these are considered separately in the analysis (as seen in Fig. 4D).

### Generation of P-P correlation plots

To visualize correlation between P_GWAS_ and P_eQTL_, each variant within the LOI is plotted with P_eQTL_ along the horizontal axis, and P_GWAS_ along the vertical axis. Correlation between the two probabilities is visualized by plotting a best-fit linear regression over the points. The Pearson correlation coefficient (r) and *p*-value of correlation (p) are computed and displayed on the plot as well (Fig. 1D, 2D). If an analysis differentiating between congruous and incongruous variants is specified, separate plots are made for each set of variants and superimposed over each other as a single plot, with linear regression lines/Pearson coefficients displayed for both sets.

If LD data is supplied in the form of **LD.df**, a similar plot is generated, but the fill color of each point is set to correspond to the LD R^2^ value for each variant with a specified lead variant, plotted as a green diamond (Fig. 3E). This lead variant can be user-specified with the argument **leadSNP** or is otherwise automatically defined as the upper-right-most variant in the P-P plot. This same lead variant is also labelled in the main eQTpLot panel A (Fig. 3A). In the case where LD data is provided and an analysis differentiating between congruous and incongruous variants is specified, two separate plots are generated: one for congruous and one for incongruous variants (Fig. 4E-F). In each plot, the fill color of each point is set to correspond to the LD R^2^ value for each variant with the lead variant for that specific plot (again defined as the upper-right most variant of the P-P plot), with both the congruous and incongruous lead variants labelled in the main eQTpLot panel A (Fig. 4A).

## Use examples

To more clearly illustrate the use and utility of the eQTpLot software, the following 3 examples are provided. In example 1, the basic implementation of eQTpLot illustrates a plausible candidate gene, *BBS1*, for a GWAS association peak for LDL cholesterol on chromosome 11, while also illustrating the colocalization between the GWAS signal and eQTL data for a different, less plausible candidate gene at the same locus, *ACTN3*. In example 2 the *BBS1* gene is further investigated through the use of the TissueList function, and through the inclusion of LD data into the eQTpLot analysis. Lastly, in example 3, the visualization is further refined by differentiating between variants with congruous and incongruous directions of effect on *BBS1* expression levels and the LDL cholesterol trait.

### Example 1 – comparing eQTpLots for two genes within a linkage peak

A GWAS study of LDL cholesterol levels has identified a significant association with a genomic locus at chr11:66,196,265- 66,338,300 (build hg19), which contains a number of plausible candidate genes, including *BBS1* and *ACTN3*. eQTpLot is employed in R to illustrate eQTL colocalization for the *BBS1* and *ACTN3* genes and the LDL cholesterol signal as follows.

Using the **GeneList** function of eQTpLot, the user supplies both the *BBS1* and *ACTN3* genes to eQTpLot, along with all required input data, to obtain a crude estimation of which gene’s eQTL data most closely correlates with the GWAS signal observed at this locus. Calling eQTpLot as follows:

$$ \mathsf{eQTpLot}\ \left(\mathsf{GWAS}.\mathsf{df}=\mathsf{gwas}.\mathsf{df}.\mathsf{example},\mathsf{eQTL}.\mathsf{df}=\mathsf{eqtl}.\mathsf{df}.\mathsf{example},\mathsf{gene}=\mathsf{c}\left("\mathsf{BBS1}","\mathsf{ACTN3}"\right),\mathsf{gbuild}="\mathsf{hg}\mathsf{19}",\mathsf{trait}="\mathsf{LDL}",\mathsf{tissue}="\mathsf{all}",\mathsf{CollapseMethod}="\mathsf{\min}",\mathsf{GeneList}=\mathsf{T}\right) $$

eQpLot generates Pearson correlation statistics between P_GWAS_ and P_eQTL_ for both genes and the LDL trait, using a PanTissue approach (collapsing by method “min” as described above). The output generated is:

$$ \mathsf{eQTL}\ \mathsf{analysis}\ \mathsf{for}\ \mathsf{gene}\ \mathsf{BBS1}:\mathsf{Pearson}\ \mathsf{correlation}:\mathsf{0.823},\mathit{\mathsf{p}}-\mathsf{value}:\mathsf{1.62}\mathsf{e}-\mathsf{127}\mathit{\mathsf{e}\mathsf{QTL}\ \mathsf{analysis}\ \mathsf{for}\ \mathsf{gene}\ \mathsf{ACTN3}}:\mathit{\mathsf{Pearson}\ \mathsf{correlation}}:\mathit{\mathsf{0.245}},\mathit{\mathsf{p}}-\mathit{\mathsf{value}}:\mathit{\mathsf{1.52}}\mathit{\mathsf{e}}-\mathit{\mathsf{07}} $$

Demonstrating that there is significantly stronger correlation between the GWAS signal at this locus and eQTLs for the gene *BBS1*, compared to the gene *ACTN3*. To visualize these differences using eQTpLot, starting with the gene *BBS1*, eQTpLot can be called as follows:

$$ \mathsf{eQTpLot}\ \left(\mathsf{GWAS}.\mathsf{df}=\mathsf{gwas}.\mathsf{df}.\mathsf{example},\mathsf{eQTL}.\mathsf{df}=\mathsf{eqtl}.\mathsf{df}.\mathsf{example},\mathsf{gene}="\mathsf{BBS1}",\mathsf{gbuild}="\mathsf{hg}\mathsf{19}",\mathsf{trait}="\mathsf{LDL}",\mathsf{tissue}="\mathsf{all}",\mathsf{CollapseMethod}="\mathsf{\min}"\right) $$

As written, this command will analyze the GWAS data, as contained within GWAS.df.example, within a default 200 kb range surrounding the *BBS1* gene, using the preloaded **Genes.df** to define the genomic boundaries of *BBS1* based on genome build hg19. eQTL data from eQTL.df.example will be filtered to contain only data pertaining to *BBS1*. Since **tissue** is set to “all,” eQTpLot will perform a PanTissue analysis, as described above.

The resulting plot (Fig. 1) illustrates clear evidence of colocalization between the LDL-significant locus and *BBS1* eQTLs. In Fig. 1A, it is easy to see that all variants significantly associated with LDL cholesterol (those plotted above the horizontal red line) are also very significantly associated with *BBS1* expression levels, as indicated by their coloration in bright orange. Figure 1C shows that there is a significant enrichment (*p* = 9.5e-46 by Fisher’s exact test) for *BBS1* eQTLs among GWAS-significant variants. Lastly, Fig. 1D illustrates strong correlation between P_GWAS_ and P_eQTL_ for the analyzed variants, with a Pearson correlation coefficient of 0.823 and a *p*-value of correlation of 1.62e-127 (as displayed on the plot). Taken together, these analyses provides strong evidence for colocalization between variants associated with LDL cholesterol levels and variants associated with *BBS1* expression levels at this genomic locus.

To visualize the possibility that the LDL association signal might also be acting through modulation of the expression of *ACTN3* at this locus, the same analysis can be performed, substituting the gene *ACTN3* for the gene *BBS1*, as in the following command:

$$ \mathsf{eQTpLot}\ \left(\mathsf{GWAS}.\mathsf{df}=\mathsf{GWAS}.\mathsf{df}.\mathsf{example},\mathsf{eQTL}.\mathsf{df}=\mathsf{eQTL}.\mathsf{df}.\mathsf{example},\mathsf{gene}="\mathsf{ACTN3}",\mathsf{gbuild}="\mathsf{hg}\mathsf{19}",\mathsf{trait}="\mathsf{LDL}",\mathsf{tissue}="\mathsf{all}",\mathsf{CollapseMethod}="\mathsf{\min}"\right) $$

Unlike the previous example, the resultant plot (Fig. 2) illustrates poor evidence for colocalization between *ACTN3* eQTLs and LDL cholesterol-significant variants. Although there is significant enrichment for *ACTN3* eQTLs among GWAS-significant variants (Fig. 2B), there is poor evidence for correlation between P_GWAS_ and P_eQTL_ (Fig. 2D), and it is intuitively clear in Fig. 2A that the eQTL and GWAS signals do not colocalize (the brightest colored points with the strongest association with *ACTN3* expression are not among the variants most significantly associated with LDL cholesterol levels).

### Example 2 – the TissueList function and adding LD information to eQTpLot

The plots generated in Example 1 illustrated colocalization between *BBS1* eQTLs and the GWAS peak for LDL cholesterol on chromosome 11, using a PanTissue analysis approach. The user may next wish to investigate if there are specific tissues in which *BBS1* expression is most clearly correlated with the LDL GWAS peak. Using the **TissueList** function of eQTpLot as follows:

$$ \mathsf{eQTpLot}\ \left(\mathsf{GWAS}.\mathsf{df}=\mathsf{gwas}.\mathsf{df}.\mathsf{example},\mathsf{eQTL}.\mathsf{df}=\mathsf{eqtl}.\mathsf{df}.\mathsf{example},\mathsf{gene}="\mathsf{BBS1}",\mathsf{gbuild}="\mathsf{hg}\mathsf{19}",\mathsf{trait}="\mathsf{LDL}",\mathsf{tissue}="\mathsf{all}",\mathsf{TissueList}=\mathsf{T}\right) $$

eQTpLot generates Pearson correlation statistics between P_GWAS_ and P_eQTL_ for *BBS1* and the LDL trait across each tissue contained within eQTL.df. The resultant output, ranked by degree of correlation, is as follows

$$ \mathsf{eQTL}\ \mathsf{analysis}\ \mathsf{for}\ \mathsf{tissue}\ \mathsf{Cells}\_\mathsf{Cultured}\_\mathsf{fibroblasts}:\mathsf{Pearson}\ \mathsf{correlation}:\mathsf{0.902},\mathit{\mathsf{p}}-\mathsf{value}:\mathsf{1.12}\mathsf{e}-\mathsf{65}\mathit{\mathsf{e}\mathsf{QTL}\ \mathsf{analysis}\ \mathsf{for}\ \mathsf{tissue}\ \mathsf{Whole}}\_\mathit{\mathsf{Blood}}:\mathit{\mathsf{Pearson}\ \mathsf{correlation}}:\mathit{\mathsf{0.85}},\mathit{\mathsf{p}}-\mathit{\mathsf{value}},\mathit{\mathsf{1.64}}\mathit{\mathsf{e}}-\mathit{\mathsf{55}}\mathit{\mathsf{e}\mathsf{QTL}\ \mathsf{analysis}\ \mathsf{for}\ \mathsf{tissue}\ \mathsf{Brain}}\_\mathit{\mathsf{Frontal}}\_\mathit{\mathsf{Cortex}}\_\mathit{\mathsf{BA9}}:\mathit{\mathsf{Pearson}\ \mathsf{correlation}},\mathit{\mathsf{0.84}},\mathit{\mathsf{p}}-\mathit{\mathsf{value}}:\mathit{\mathsf{1.02}}\mathit{\mathsf{e}}-\mathit{\mathsf{51}}\mathit{\mathsf{e}\mathsf{QTL}\ \mathsf{analysis}\ \mathsf{for}\ \mathsf{tissue}\ \mathsf{Brain}}\_\mathit{\mathsf{Nucleus}}\_\mathit{\mathsf{accumbens}}\_\mathit{\mathsf{basal}}\_\mathit{\mathsf{ganglia}}:\mathit{\mathsf{Pearson}\ \mathsf{correlation}}:\mathit{\mathsf{0.84}\mathsf{1}},\mathit{\mathsf{p}}-\mathit{\mathsf{value}}:\mathit{\mathsf{1.74}}\mathit{\mathsf{e}}-\mathit{\mathsf{48}}\mathit{\mathsf{e}\mathsf{QTL}\ \mathsf{analysis}\ \mathsf{for}\ \mathsf{tissue}\ \mathsf{Brain}}\_\mathit{\mathsf{Cortex}}:\mathit{\mathsf{Pearson}\ \mathsf{correlation}}:\mathit{\mathsf{0.818}},\mathit{\mathsf{p}}-\mathit{\mathsf{value}}:\mathit{\mathsf{2.44}}\mathit{\mathsf{e}}-\mathit{\mathsf{43}}\mathit{\mathsf{e}\mathsf{QTL}\ \mathsf{analysis}\ \mathsf{for}\ \mathsf{tissue}\ \mathsf{Esophagus}}\_\mathit{\mathsf{Gastroesophageal}}\_\mathit{\mathsf{Junction}}:\mathit{\mathsf{Pearson}\ \mathsf{correlation}}:\mathit{\mathsf{0.85}\mathsf{2}},\mathit{\mathsf{p}}-\mathit{\mathsf{value}}:\mathit{\mathsf{2.15}}\mathit{\mathsf{e}}-\mathit{\mathsf{23}}\mathit{\mathsf{e}\mathsf{QTL}\ \mathsf{analysis}\ \mathsf{for}\ \mathsf{tissue}\ \mathsf{Skin}}\_\mathit{\mathsf{Sun}}\_\mathit{\mathsf{Exposed}}\_\mathit{\mathsf{Lower}}\_\mathit{\mathsf{leg}}:\mathit{\mathsf{Pearson}\ \mathsf{correlation}}:\mathit{\mathsf{0.562}},\mathit{\mathsf{p}}-\mathit{\mathsf{value}}:\mathit{\mathsf{1.52}}\mathit{\mathsf{e}}-\mathit{\mathsf{21}}. $$

*…*

This output demonstrates a strong correlation between LDL cholesterol levels and *BBS1* expression levels in a number of tissues. To further explore these associations, the user can specifically run eQTpLot on data from a single tissue, for example Whole_Blood, while also supplying LD data to eQTpLot using the argument **LD.df**:

$$ \mathsf{eQTpLot}\ \left(\mathsf{GWAS}.\mathsf{df}=\mathsf{GWAS}.\mathsf{df}.\mathsf{example},\mathsf{eQTL}.\mathsf{df}=\mathsf{eQTL}.\mathsf{df}.\mathsf{example},\mathsf{gene}="\mathsf{BBS1}",\mathsf{gbuild}="\mathsf{hg}\mathsf{19}",\mathsf{trait}="\mathsf{LDL}",\mathsf{tissue}="\mathsf{Whole}\_\mathsf{Blood}",\mathsf{LD}.\mathsf{df}=\mathsf{LD}.\mathsf{df}.\mathsf{example},\mathsf{R}\mathsf{2}\mathsf{\min}=\mathsf{0.25},\mathsf{LD}\mathsf{min}=\mathsf{100}\right) $$

Here the argument **LD.df** refers to the LD.df.example data frame containing a list of pairwise LD correlation measurements between all the variants within the LOI, as one might obtain from a PLINK linkage disequilibrium analysis using the --r2 option [10]. Additionally, the parameter **R2min** is set to 0.25, indicating that **LD.df **should be filtered to drop variant pairs in LD with R^2^ less than 0.25. **LDmin** is set to 100, indicating that only variants in LD with at least 100 other variants should be plotted in the LD heatmap.

The resultant plot, Fig. 3, is different than Fig. 1 in two important ways. First, a heat map of the LD landscape for all *BBS1* cis-eQTL variants in Whole_Blood within the LOI is shown in Fig. 3C; this heatmap makes it clear that a number of *BBS1* eQTL variants are in strong LD with each other at this locus. Second, the P-P plot, Fig. 3E, now includes LD information for all plotted variants; a lead variant, rs3741360, has been defined (by default the upper-right most variant on the P-P plot), and all other variants are plotted with a color scale corresponding to their squared coefficient of linkage correlation with this lead variant. eQTpLot also labels the lead variant in Fig. 3A for reference. With the incorporation of this new data, we can now see that most, but not all, of the GWAS-significant variants are in strong LD with each other. This implies that there are at least two distinct LD blocks at the *BBS1* locus with strong evidence of colocalization between the *BBS1* eQTL and LDL GWAS signals.

### Example 3 – separating congruous from incongruous variants

In addition to including LD data in our eQTpLot analysis, we can also include information on the directions of effect of each variant, with respect to the GWAS trait and *BBS1* expression levels. This is accomplished by setting the argument **congruence **to TRUE:

$$ \mathsf{eQTpLot}\ \left(\mathsf{GWAS}.\mathsf{df}=\mathsf{GWAS}.\mathsf{df}.\mathsf{example},\mathsf{eQTL}.\mathsf{df}=\mathsf{eQTL}.\mathsf{df}.\mathsf{example},\mathsf{gene}="\mathsf{BBS1}",\mathsf{gbuild}="\mathsf{hg}\mathsf{19}",\mathsf{trait}="\mathsf{LDL}",\mathsf{tissue}="\mathsf{Whole}\_\mathsf{Blood}",\mathsf{LD}.\mathsf{df}=\mathsf{LD}.\mathsf{df}.\mathsf{example},\mathsf{R}\mathsf{2}\mathsf{\min}=\mathsf{0.25},\mathsf{LD}\mathsf{min}=\mathsf{100},\mathsf{congruence}=\mathsf{TRUE}\right) $$

The resulting plot, Fig. 4, divides all *BBS1* eQTL variants in Whole_Blood into two groups: congruent – those variants associated with either an increase in both, or decrease in both *BBS1* expression levels and LDL levels – and incongruent – those variants with opposite directions of effect on *BBS1* expression levels and LDL levels. In carrying out such an analysis, it becomes clear that it is specifically variants with congruent directions of effect that are driving the signal colocalization; that is, variants associated with decreases in *BBS1* expression strongly colocalize with variants associated with decreases in LDL cholesterol.

## Conclusions

eQTpLot provides a unique, user-friendly, and intuitive means of visualizing cis-eQTL and GWAS signal colocalization in a single figure. As plotted by eQTpLot, colocalization between GWAS and eQTL data for a given gene-trait pair is immediately visually obvious, and can be compared across candidate genes to quickly generate hypotheses about the underlying causal mechanisms driving GWAS association peaks. Additionally, eQTpLot allows for Pan- and MultiTissue eQTL analysis, and for the differentiation between eQTL variants with congruous and incongruous directions of effect on GWAS traits – two features not found in any other visualization software. We believe eQTpLot will prove a useful tool for investigators seeking a convenient and customizable visualization of eQTL and GWAS data colocalization.

## Availability and requirements

**Project name:** eQTpLot

**Project home page:** https://github.com/RitchieLab/eQTpLot

**Operating system(s):** Platform independent

**Programming language:** R

**Other requirements:** None

**License:** GNU GPL

**Any restrictions to use by non-academics:** None.

## Data Availability

The eQTpLot R package and tutorial, along with the necessary datasets to generate the four example plots discussed in this manuscript, are available at. https://github.com/RitchieLab/eQTpLot. The eQTL data used to generate the eQTL.df file were generated previously, and are freely available through the GTEx Portal [11]. The GWAS summary statistics used to generate the GWAS.df file used in this manuscript are available at https://ritchielab.org/publications/supplementary-data/ajhg-cilium and are based on a study utilizing data available through the UK Biobank (UKBB) [24, 25]. As a part of our agreement to use the data contained within UKBB, we are not allowed to share the raw data ourselves, but individuals who are interested can request access.

## Abbreviations

eQTL: Expression Quantitative Trait Loci
GWAS: Genome-wide Association Study
LD: Linkage disequilibrium
LOI: Locus of Interest
NES: Normalized effect size
P_GWAS_: *p*-value of a given variant’s association with a GWAS trait
P_eQTL_: p-value of a given variant’s association with a gene’s expression levels
R^2^: the squared coefficient of linkage correlation between two variants
